# Treat-and-Extend vs. Pro Re Nata Regimen of Ranibizumab for Diabetic Macular Edema—A Two-Year Matched Comparative Study

**DOI:** 10.3389/fmed.2021.781421

**Published:** 2022-01-25

**Authors:** Tso-Ting Lai, Ta-Ching Chen, Chang-Hao Yang, Chung-May Yang, Tzyy-Chang Ho, Yi-Ting Hsieh

**Affiliations:** ^1^Department of Ophthalmology, National Taiwan University Hospital, Taipei, Taiwan; ^2^College of Medicine, Graduate Institute of Clinical Medicine, National Taiwan University, Taipei, Taiwan; ^3^College of Medicine, National Taiwan University, Taipei, Taiwan

**Keywords:** diabetic macular edema, ranibizumab, anti-VEGF (vascular endothelial growth factor), pro re nata (PRN), treat-and-extend (T&E)

## Abstract

**Purpose:**

To compare 2-year treatment outcomes of ranibizumab using treat-and-extend (T&E) or pro re nata (PRN) regimens for diabetic macular edema (DME) in clinical settings.

**Methods:**

We retrospectively enrolled 34 patients (34 eyes) with DME treated with ranibizumab using the T&E regimen, and 34 patients (34 eyes) treated with ranibizumab using the PRN regimen and matched to cases in the treat-and-extend group by baseline best-corrected visual acuity (BCVA) and central foveal thickness (CFT). BCVA and CFT changes, number of injections and recurrence of macular edema over 2 years were compared between the groups.

**Results:**

The average BCVA gain in the T&E and PRN groups was 16.2 and 7.6 ETDRS letters at 2 years (*p* = 0.011), respectively. The mean CFT reduction was 145.5 ± 127.3 and 97.3 ± 152.5 μm in the T&E and PRN groups at 2 years (*p* = 0.035), respectively. The T&E group had a higher proportion of patients with BCVA gain ≥ 15 letters at months 18 (*p* = 0.015) and 24 (*p* = 0.029) than the PRN group. During the 2-year treatment periods, the T&E group received more injections than the PRN group (11.0 ± 3.2 vs. 6.2 ± 2.0; *p* < 0.001), while the PRN group had more recurrence of macular edema than the T&E group (71 vs. 41%; *p* = 0.015).

**Conclusions:**

After 2-year ranibizumab treatment for DME, better visual and anatomical improvement and less recurrence of macular edema were achieved in the T&E group, with more injections administered.

## Introduction

Currently, the first-line treatment for diabetic macular edema (DME), a condition that leads to severe visual impairment in 28–29% of patients with diabetes mellitus (1), is anti-vascular endothelial growth factor (VEGF) therapy (2). The superior effectiveness over laser photocoagulation in improving visual acuity and reducing edema in DME has been demonstrated in several clinical trials (3–7). With various treatment protocols applied in different studies, patients were able to gain 6.1–10.3 Early Treatment Diabetic Retinopathy Study (ETDRS) letters of vision in 1 year. Furthermore, visual benefits could be maintained after 4–5 years with continuous anti-VEGF injections (8, 9). However, the favorable outcomes from these clinical trials did not always translate into similar results in clinical practice settings. Globally, real-world clinical practice studies have reported poorer visual improvements along with the use of fewer injections than reported in clinical trials (10–14). The need for frequent follow-up visits and repeated injections, along with other factors, leads to poor compliance in patients with DME and is responsible for the inferior outcomes in real-world studies (14).

Different treatment protocols for anti-VEGF therapy have been developed in recent years to optimize the treatment effects and cost effectiveness. A fixed dosing regimen, either monthly or bimonthly treatment after a loading phase, was proven to be effective in phase III registrational trials, such as RISE, RIDE (7), VIVID and VISTA (15), but was difficult to follow in real-world clinical practice. An “as needed” or *pro re nata* (PRN) approach was developed to decrease the number of injections while maintaining a fixed follow-up schedule to closely monitor treatment responses. Clinical trials using the PRN regimen reduced the mean injections to 7–10 in the first year with monthly monitoring (3, 5, 6, 16). A treat-and-extend (T&E) regimen, different from other protocols, involved gradual increase in duration between each follow-up visit once the patient achieved a preset “stable” condition along with an injection administered at every visit (17). The RETAIN study (18) first reported the use of the T&E protocol for patients with DME and found a similar visual improvement as that with the PRN regimen, while reducing 46% of clinic visits. The TREX-DME study (19) further demonstrated similar visual and anatomical improvements in both T&E and monthly dosing groups in their 2-year results, with significantly reduced injections using their T&E algorithm. The sustainable efficiency of the T&E regimen and its ability to reduce treatment burden shown in clinical trials may further benefit patients in real-world conditions. However, there is limited evidence regarding the use of the T&E protocol in DME in clinical settings.

Herein, we compared the 2-year real-world visual and anatomical outcomes of patients with DME treated with either the T&E or PRN regimen to better understand their efficacy in clinical practice setting.

## Methods

### Study Population and Setting

In this matched comparative study, we retrospectively reviewed all patients who received their first anti-VEGF injection for DME at the National Taiwan University Hospital between November 2014 and November 2016. Patients who received intravitreal injection (IVI) of 0.5 mg ranibizumab (Lucentis®, Genentech, San Francisco, CA/Norvatis, Basel, Switzerland) following a T&E regimen and who were followed up for at least 2 years were included in the study (T&E) group. The same number of patients who received ranibizumab injection using a PRN protocol during the same period and were matched to the cases in the T&E group by baseline best-corrected visual acuity (BCVA) (difference ≤ 1 line) and baseline central foveal thickness (CFT) (difference ≤ 10%) on optical coherence tomography (OCT), were randomly selected and included in the control (PRN) group. The other inclusion criteria were as follows: baseline Snellen BCVA between 20/400 and 20/40, baseline CFT above 300 μm, and evidence of DME on fluorescence angiography (FA) without other causes of macular edema. Patients who had received anti-VEGF therapy at another hospital or for other etiologies within 6 months prior to the first injection at our hospital were excluded. Patients who underwent intraocular surgery other than IVI (such as cataract surgery or vitrectomy) during the study period and those who used different anti-VEGF agents during the follow-up period were also excluded. This study adhered to the tenets of the Declaration of Helsinki. The National Taiwan University Hospital Research Ethics Committee approved the study (No.: 201811023RIFD), and waiver of informed consent was obtained due to its retrospective nature.

### Treatment Protocols

All patients received 3-monthly loading injections of ranibizumab. After the loading phase, patients in the T&E group could extend their follow-up and treatment visits if they had no disease activity on OCT images and the BCVA was either improved or stable compared to that at the last visit. The follow-up interval was extended by 4 weeks each time, starting from 4 weeks at baseline. The longest allowed follow-up interval was 24 weeks. An injection was administered at each visit after the BCVA was measured and the OCT image was acquired. The follow-up interval was shortened by 4 weeks if OCT revealed new disease activity, with a minimum of 4 weeks between each visit.

In the PRN group, the patients received monthly injections after the loading phase until the OCT image showed no disease activity and the BCVA was either improved or stable compared to that at the last visit. The patients then received regular follow-up, usually every 1–3 months, as determined by the treating physician and the patient, and received no further injection unless recurrence of disease activity was noted on OCT during follow-up.

Disease activity on OCT was defined as fluid accumulation (either intraretinal or subretinal) with a CFT > 300 μm. For patients with persistent disease activity after the loading treatment, the physicians might add supplementary treatments, including macular laser or subtenon injection of triamcinolone acetonide. For patients with decreased but persistent disease activity after 5 monthly injections of ranibizumab, we extended the treatment interval (in the T&E group) or discontinued the treatment (in the PRN group). Details of the treatment protocols are shown in Supplementary Table 1.

### Clinical Data Collection

Baseline demographic data, including age, sex, serum HbA1c, and previous treatments such as focal/grid laser, steroid injections, panretinal photocoagulation, and anti-VEGF injection, were recorded. The BCVA was converted to the logarithm of the minimum angle of resolution (logMAR) score for calculation, and the change in BCVA was converted to a number of ETDRS letters. All patients underwent FA examination at baseline, and the images were reviewed independently by two retinal specialists (TTL and YTH) for the presence of proliferative diabetic retinopathy. All OCT images at baseline were reviewed by the two investigators for the presence of epiretinal membrane (ERM), intraretinal cyst (IRC), subretinal fluid (SRF), hyperreflective foci (HF), ellipsoid zone (EZ) disruption, and disorganization of the retinal inner layers (DRIL). The CFT was measured using the central 1-mm thickness built-in thickness map program of RTVue OCT (Optovue, Inc., Fremont, CA). The numbers of injections and clinic visits for each patient in the first and second years were recorded. The number of recurrences of DME, defined as disease activity on OCT leading to shortening of the treatment interval in the T&E group or restart of IVI in the PRN group, was also documented.

### Statistical Analysis

The BCVA and CFT at preset time points (at baseline and at 3, 6, 12, 18, and 24 months after the first injection) were evaluated, with the last observation carried forward method used for any missing data because of the individualized follow-up schedule. For comparison between the T&E and PRN groups, we used paired *t*-tests for all continuous variables and Fisher's exact tests for all categorical variables. The proportions of patients with BCVA gain ≥ 15 letters or with visual loss ≥ 5 letters at each time point were compared between the two groups. The comparison of BCVA and CFT between baseline and different follow-up time points for individual patients was performed using paired *t*-tests. Factors associated with the final BCVA improvement were analyzed using linear regression. Baseline BCVA, baseline OCT biomarkers (CFT, IRC, SRF, HF, DRIL, and EZ disruption), treatment protocol, total number of injections, and recurrence of macular edema were included in the univariate analysis with adjustment for age and baseline BCVA and CFT. Factors that were significantly associated with final BCVA improvement in the univariate analysis were then included in the multivariate linear regression using the stepwise approach. Data were analyzed using SPSS software (SPSS 22.0; SPSS Inc., Chicago, IL, USA). Statistical significance was set at *p* < 0.05.

## Results

### Baseline Characteristics

Thirty-four eyes of 34 patients with DME who received ranibizumab injection using a T&E protocol were included in the study group, and 34 eyes of 34 patients treated under a PRN protocol were included in the control group. The baseline demographics and OCT findings of the two groups are summarized in Table 1. The two groups were matched for the baseline BCVA and CFT, and there were no differences in age, sex, previous treatments, or severity of diabetic retinopathy between the two groups. As for OCT biomarkers, the proportions of patients with ERM, IRC, SRF, HF, and EZ disruption at baseline were similar in both groups, except that more patients in the PRN group had DRIL at baseline.

**Table 1:** Baseline characteristics and optical coherence tomographic findings of patients with diabetic macular edema who underwent anti-vascular endothelial growth factor therapy using pro re nata or treat-and-extend protocol.

	**PRN 34 eyes**	**T&E 34 eyes**	***P*-value**
Age (years, mean ± SD)	62.3 ± 7.3	60.6 ± 8.8	0.377
Sex (M:F)	21:13	17:17	0.329
HbA1c (mean±SD)	7.54 ± 1.10	7.42 ± 1.18	0.677
Pseudophakic (No./%)	5 (14.7)	8 (23.5)	0.355
PDR (No./%)	14 (41.2)	17 (50.0)	0.465
PRP (No./%)	12 (35.3)	11 (32.4)	0.798
Previous anti-VEGF (No./%)	7 (20.6)	6 (17.6)	0.758
Previous non-anti-VEGF treatment (No./%)	3 (8.8)	5 (14.7)	0.709
Preoperative BCVA (logMAR, mean±SD)	0.710 ± 0.310	0.723 ± 0.333	0.487
**Baseline OCT features**			
CFT (μm, mean ± SD)	446.2 ± 126.7	438.2 ± 119.2	0.629
ERM (No./%)	9 (26.5)	5 (14.7)	0.369
IRC (No./%)	33 (91.1)	31 (91.2)	0.614
SRF (No./%)	12 (35.3)	9 (26.5)	0.431
DRIL (No./%)	24 (70.6)	10 (29.4)	0.001
HF (No./%)	31 (91.2)	30 (88.2)	1.000
EZ disruption (No./%)	20 (58.8)	15 (44.1)	0.225

*BCVA, best-corrected visual acuity; CFT, central foveal thickness; DRIL, disorganization of retinal inner layers; ERM, epiretinal membrane; EZ, ellipsoid zone; HF, hyperreflective foci; IRC, intraretinal cyst; logMAR, logarithm of the minimum angle of resolution; OCT, optical coherence tomography; PDR, proliferative diabetic retinopathy; PRN, pro re nata; PRP, panretinal photocoagulation; SD, standard deviation; SRF, subretinal fluid; T&E, treat-and-extend; VEGF, vascular endothelial growth factor*.

### Visual Outcomes

**Figure 1:**
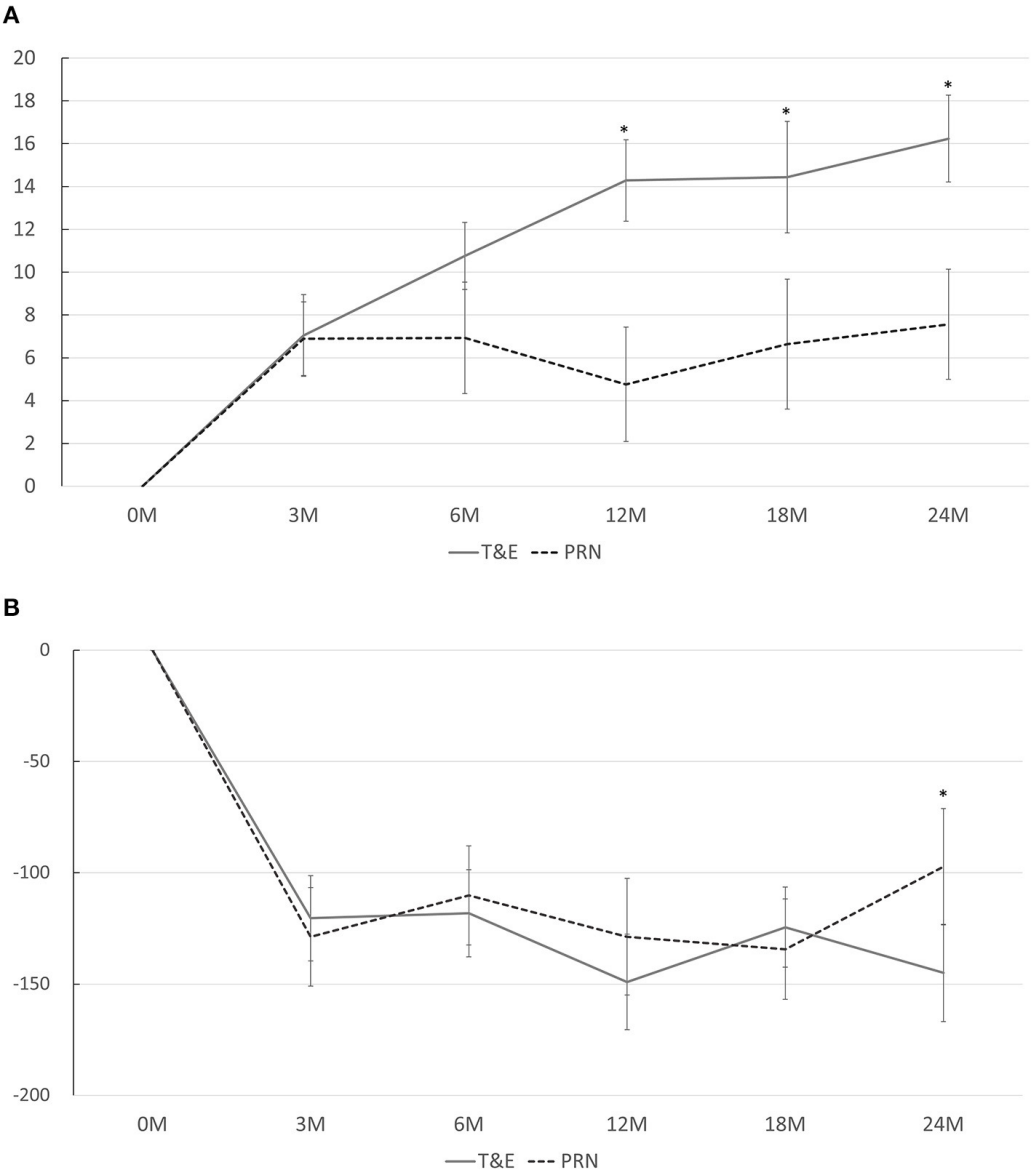
**(A)** Mean changes in the best-corrected visual acuity from baseline at different time points. A significant difference between the T&E and PRN groups is found at 12, 18, and 24 months. **(B)** Mean changes in the central foveal thickness from baseline at different time points. A significant difference between the T&E and PRN groups is found at 24 months. T&E, treat-and-extend; PRN, pro re nata. *p < 0.05.

In both groups, the BCVA improved significantly at months 3, 6, 12, 18, and 24 compared to that at the baseline, except for a borderline significance at month 12 in the PRN group (*p* < 0.001 at every time point in the T&E group; *p* < 0.001, *p* = 0.012, 0.084, 0.036, and 0.006 in the PRN group at 3, 6, 12, 18, and 24 months, respectively). The average BCVA was significantly better in the T&E group at month 12 (0.437 ± 0.247 [95% CI, 0.351–0.524]) and month 24 (0.398 ± 0.294 [95% CI, 0.296–0.501]) than in the PRN group (month 12: 0.615 ± 0.386 [95% CI, 0.481–0.750], *p* = 0.005; month 24: 0.559 ± 0.417 [95% CI, 0.414–0.704], *p* = 0.021; Table 2). The mean changes in BCVA at different time points are shown in Figure 1A. The average BCVA gains were 7.0, 10.8, 14.3, 14.4, and 16.2 ETDRS letters in the T&E group at months 3, 6, 12, 18, and 24, respectively, which showed a continuous increase from month 3 to month 24. On the other hand, the BCVA gains remained constant from month 3 to month 24 in the PRN group (6.9, 6.9, 4.8, 6.6, and 7.6 letters at months 3, 6, 12, 18, and 24, respectively), and were significantly lower than those in the T&E group at 12, 18, and 24 months (*p* = 0.003, 0.013, and 0.011, respectively).

**Table 2:** Treatment outcomes of patients with diabetic macular edema who underwent anti-vascular endothelial growth factor therapy using ranibizumab with pro re nata or treat-and-extend protocol.

	**PRN 34 eyes**	**T&E 34 eyes**	***P*-value**
**OPD visits (mean** **±SD)**			
Year 1	6.9 ± 2.0	7.4 ± 1.7	0.270
Year 2	5.8 ± 1.6	5.5 ± 1.8	0.560
Total	12.7 ± 2.6	12.9 ± 3.1	0.782
**Injection numbers (mean** **±SD)**			
Year 1	4.9 ± 1.5	7.6 ± 1.8	<0.001
Year 2	1.3 ± 1.2	3.5 ± 1.9	<0.001
Total	6.2 ± 2.0	11.0 ± 3.2	<0.001
Recurrence of macular edema (No./%)	24 (70.6)	14 (41.2)	0.015
VH (No./%)	5 (14.7)	3 (8.8)	0.709
BCVA at 12 months (logMAR, mean ± SD)	0.615 ± 0.386	0.437 ± 0.247	0.005
BCVA at 24 months (logMAR, mean ± SD)	0.559 ± 0.417	0.398 ± 0.294	0.021
CFT at 12 months (μm, mean ± SD)	321.8 ± 151.8	289.1 ± 65.1	0.287
CFT at 24 months (μm, mean ± SD)	348.9 ± 141.1	293.2 ± 62.7	0.019

*BCVA, best corrected visual acuity; CFT, central foveal thickness; logMAR, logarithm of the minimum angle of resolution; OPD, outpatient clinic; PRN, pro re nata; SD, standard deviation; T&E, treat-and-extend; VH, vitreous hemorrhage*.

**Figure 2:**
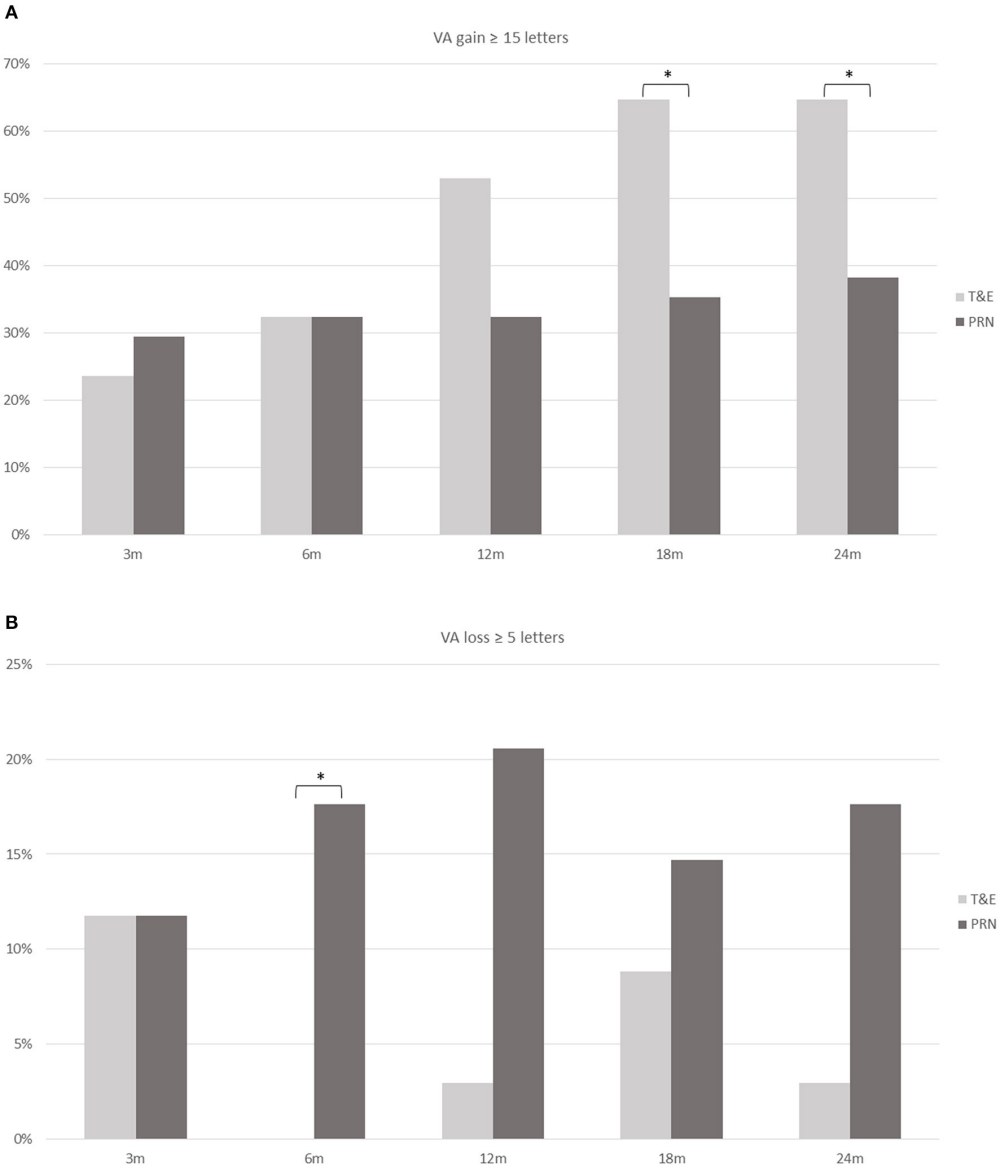
The proportion of patients who achieved a gain of ≥ 15 ETDRS letters **(A)** and those who had a loss of ≥ 5 letters **(B)** at different time points in the T&E and PRN groups. A significant difference (asterisk) is found at 18 and 24 months in those who gained ≥ 15 letters and at 6 months in those who had a loss of ≥ 5 letters. ETDRS, Early Treatment Diabetic Retinopathy Study; T&E, treat-and-extend; PRN, pro re nata; VA, visual acuity. *p < 0.05.

Figure 2 shows the proportions of patients with VA gain ≥ 15 letters and those with VA loss ≥ 5 letters. The T&E group had a higher proportion of patients with VA gain ≥ 15 letters at months 18 (*p* = 0.015) and 24 (*p* = 0.029) than the PRN group. On the other hand, a lower proportion of patients in the T&E group had VA loss ≥ 5 letters at month 6 than those in the PRN group (*p* = 0.025).

### Anatomical Outcomes

The CFTs at months 12 and 24 in both groups are shown in Table 2. The CFT was significantly lower in the T&E group than in the PRN group at month 24 (*p* = 0.019) but not at month 12 (*p* = 0.287). While the CFT in each group significantly reduced at every time point compared to that at the baseline, no difference was found between the CFT reductions in both groups at each time point except for month 24 (*p* = 0.035; Figure 1B). Six patients (17.6%) in the T&E group and 7 patients (20.6%) in the PRN groups had decreased but persistent disease activity after five consecutive monthly injections (*p* = 0.758). During the study period, 14 (41%) patients in the T&E group and 24 (71%) in the PRN group experienced at least one episode of recurrence of macular edema (*p* = 0.015). Furthermore, three (9%) patients in the T&E group and five (15%) in the PRN group experienced at least one episode of vitreous hemorrhage during the 2-year follow-up period (*p* = 0.709).

### Injections and Follow-Up Frequency

The number of clinic visits and injections are summarized in Table 2. In the T&E group, the average number of injections was 7.6 ± 1.8 (95% CI, 6.9–8.2) in the first year and 3.5 ± 1.9 (95% CI, 2.8–4.2) in the second year, whereas patients in the PRN group received significantly less injections in the first (4.9 ± 1.5 [95% CI, 4.4–5.4], *p* < 0.001) and second year (1.3 ± 1.2 [95% CI, 0.9–1.7], *p* < 0.001). Meanwhile, both groups had similar number of clinic visits in the first (T&E vs. PRN: 7.4 ± 1.7 [95% CI, 6.8–8.0] vs. 6.9 ± 2.0 [95% CI, 6.2–7.6], *p* = 0.236) and second year (5.5 ± 1.8 [95% CI, 4.9–6.2] vs. 5.8 ± 1.6 [95% CI, 5.3–6.3], *p* = 0.503). In the T&E group, the last injection interval was 12 weeks or longer in 21 (62%) patients, and 9 (26%) patients received injections at a 16-week interval or longer. During the 2-year study period, macular laser was performed in 4 and 3 patients in the T&E and PRN groups, respectively (*p* = 1.00); steroid was given in 10 and 4 patients in the T&E and PRN groups (*p* = 0.132), respectively.

### Factors Associated With Better Visual Improvement

In the univariate regression analysis, three factors, including worse baseline BCVA, T&E regimen, and absence of DRIL on baseline OCT, were associated with better BCVA gain at the final visit. Neither the total number of injections nor other baseline OCT biomarkers were associated with the final BCVA improvement. In the multivariate regression analysis, baseline BCVA and the treatment regimen were still significantly associated with the final BCVA improvement (*p* = 0.029 and 0.009, respectively) but not with the presence of DRIL (Table 3).

**Table 3:** Predicting factors for best corrected visual acuity improvement at Month 24 in patients with diabetic macular edema treated with pro re nata or treat-and-extend protocol.

**Factors**	Univariate regression^*^		**Multivariate regression**	
	**Coefficient**	***P*-value**	**Coefficient**	***P*-value**
Age	0.003	0.445		
Baseline BCVA	−0.299	0.010	−0.224	0.029
Baseline CFT on OCT	0.001	0.073		
IRC on OCT	0.016	0.910		
SRF on OCT	−0.017	0.822		
HF on OCT	−0.087	0.431		
DRIL on OCT	0.135	0.048	0.188	0.136
EZ disruption on OCT	0.083	0.281		
T&E (reference) vs. PRN	0.162	0.013	0.173	0.009
Total injection numbers	−0.019	0.059		
Recurrence of edema	0.103	0.143		

* *Adjustment for age, baseline BCVA, and baseline CFT*.

*BCVA, best-corrected visual acuity; CFT, central foveal thickness; DRIL, disorganization of retinal inner layers; EZ, ellipsoid zone; HF, hyperreflective foci; IRC, intraretinal cyst; OCT, optical coherence tomography; PRN, pro re nata; SRF, subretinal fluid; T&E, treat-and-extend*.

## Discussion

We conducted a retrospective matched comparative study to compare the real-world treatment outcomes of patients with DME who underwent IVI ranibizumab therapy following two different regimens: T&E and PRN protocols. While both regimens resulted in significantly improved visual acuity at 2 years, the T&E group had significantly more visual acuity gain and anatomical improvement at 2 years than the PRN group despite similar visual acuity and CFT at baseline. The proportion of patients with VA gain ≥ 15 letters at 24 months from the baseline was also higher in the T&E group than in the PRN group.

The treatment regimen for anti-VEGF therapy has evolved in recent years. In the treatment of neovascular age-related macular degeneration (nAMD), earlier trials used a fixed dosing regimen and reported favorable results (20). Subsequently, the PRN regimen, which used monthly evaluation and as needed treatment to reduce the number of injections, demonstrated non-inferior visual improvement at 1 year along with significantly fewer injections (21). Recently, clinical trials that incorporated the T&E regimen and a flexible visiting interval with injection at every visit after disease stabilization, demonstrated comparable visual outcomes between the T&E protocol and fixed monthly injections (22, 23). The non-inferior outcomes achieved by the T&E regimen and reduced number of visits and injections have led to increased popularity of the T&E approach. Results from a meta-analysis demonstrated that the T&E regimen resulted in better real-world visual outcomes compared to the PRN regimen in treating nAMD (24, 25). The success of the T&E regimen in reducing the number of injections (compared to monthly treatment) and visits (compared to PRN) as well as maintaining favorable visual outcomes has led to the application of the T&E protocol in treating DME.

The T&E regimen was first evaluated for its efficacy in treating DME in the RETAIN study, which showed comparable visual improvements in the T&E and PRN groups with a median of 12 injections in the T&E group and 10 injections in the PRN group in 24 months (18). The TREX-DME study compared the T&E and monthly injection regimens, and reported similar visual and anatomical outcomes in the two groups, while significantly fewer injections were needed in the T&E group (19). A small single-center, randomized study by Eichenbaum et al. also reported similar visual improvement after 2 years of ranibizumab injection following either monthly or T&E regimen for patients with DME (26). Despite the success of using the T&E protocol with ranibizumab in treating patients with DME in these clinical trials, there is limited evidence regarding the efficacy of T&E regimen in treating these patients under real-life conditions. A study by Ebneter et al. compared the 1-year visual outcomes of patients with DME treated using a BCVA-guided PRN regimen or an OCT-guided T&E regimen and reported similar visual gain (8.3 vs. 9.3%, respectively) at 12 months (27). A non-comparative study reported the 2-year visual outcomes of T&E protocol in patients with DME using ranibizumab, in which an average of 4.7 letters was achieved after a mean of 9.7 injections in the first year and 7.9 injections in the second year (28). In another study, a mean BCVA gain of 6.3 letters was noted at 1 year after 10 ranibizumab injections (29). In our study, greater visual improvement was observed in the T&E group than in the PRN group. The patients in the PRN group in our study received less frequent monitoring and injections compared to those in the clinical trial, which is frequently observed in real-life situations (10, 14, 30, 31). This might have led to inferior results in our patients. On the other hand, patients in the T&E group of our study had a similar number of visits yet received more injections during the study period, which resulted in better visual improvements. Beside more anti-VEGF injections, patients in the T&E group also received more subtenon steroid injection although the difference was not statistically significant. This might indicate that patients in the T&E group were treated more aggressively for DME, thus having better visual gain. In addition, visual gain in the T&E group was greater in our study than that in other real-world studies, despite fewer injections in the first and second years. The inferior baseline visual acuity might be responsible for this difference. Based on our results, we suggest the use of the T&E regimen for treating patients with DME in clinical practice.

The maximum allowed treatment intervals were usually set at 3 months in the previous studies using the T&E regimen (18, 19, 26, 28, 32–34), and 25–75% of the enrolled patients had their treatment intervals extended to 12 weeks (19, 28, 32–34). The results of our study were in line with those of previous reports, with more than half of our patients in the T&E group receiving injections every 3 months or longer at the final visit. In Protocol I study, the median number of injections was 2–3 in the second year, indicating that a significant proportion of patients might not need frequent treatment in the second year (9). Therefore, we allowed a maximum 24-week interval between the treatments. Under such a protocol, the treatment interval could be further lengthened to 16 weeks or longer in 25% of our cohort. Another study by Hirano et al. reported that 66.7% of their patients could have the treatment interval extended to 16 weeks (the maximum allowed interval) under their T&E protocol using aflibercept (35). In their study, after a mean of 11.4 injections, the average BCVA improved from 60.5 to 66.6 letters at 2 years. The inferior initial visual acuity noted in our study might indicate a more severe disease at baseline, and therefore, our patients required more frequent injections than those reported by Hirano et al. in their study. Our study results and the evidence from previous reports suggest that the maximum allowed treatment interval for patients with DME could be set at 16 weeks or longer in some patients when the T&E regimen was applied.

In the present study, the presence of DRIL at baseline was associated with less visual improvement at 2 years. Patients with diabetic retinopathy, either with or without DME, have been reported to have worse baseline visual acuity if DRIL was present on baseline OCT (36, 37). A previous study showed that DRIL was linked to vascular ischemia with poor vessel density in the inner retina (38), and patients with DME with low vessel density in the inner retina also had poorer visual improvement after resolution of macular edema (39). Furthermore, some studies reported that patients with resolved DRIL after treatment had better visual improvements (36, 40). A recent study by Zur et al. showed that patients without baseline DRIL showed better visual improvement after treatment with dexamethasone implants (41). Our study further supports the predictive value of baseline DRIL as a biomarker for poor visual improvement in patients with DME, not only in those treated with steroid implants, but also in those treated with anti-VEGF therapy.

In our study, patients in the PRN group had a lower number of injections but a higher proportion of macular edema recurrence, yet these two factors were not significantly correlated with final visual improvement in the regression analysis. Regarding the injection number, since patients with rapid response to anti-VEGF therapy in both groups may have received less injections due to improved vision and absence of disease activity, the total injection number could be affected by the aggressiveness of treatment and individual susceptibility to anti-VEGF therapy. Therefore, the total number of injection may not correlate with final visual outcome when evaluated at the individual patient level (i.e., in regression analysis). However, when the correlation was evaluated among 2 groups of patients (i.e., PRN vs. T&E), the injection number might represent the aggressiveness of treatment. As for recurrence of macular edema, it is shown that the average CFT increased at month 6 in the PRN group, while the vision deteriorated at month 12. This means that recurrence of macular edema may precede the visual deterioration, although resolution of recurrent macular edema could be achieved after further treatment with visual regain. Theoretically, frequent recurrence of macular edema may result in poor long-term visual outcomes, but this might not be significantly detected during the 2-year follow-up period. Further studies with longer follow-up periods are needed to confirm our hypothesis.

There are several limitations to our study, including the small sample size and the retrospective study design. Another concern was that the decision to apply the T&E or PRN regimen in each patient was not randomized. Although the groups of patients were matched for baseline BCVA and CFT, there is a possibility of undetected bias between the groups of patients. Additionally, the follow-up intervals in the PRN group not only were individualized and determined by the treatment response of individual patients, but might also be affected by socioeconomic factors such as the availability of frequent clinic visits. However, it was difficult to determine the exact number of visits that patients skipped their injection according to their own will due to the retrospective nature of this study. In addition, since the patients were treated under real-life condition, some patients might have received the PRN injection a few days later than the clinic visit due to the unavailability of the patients or the treating physicians. However, this just reflects the disadvantage of the PRN regimen in the clinical settings. Another limitation of the present study was that we only included patients that have completed 2 years of follow-ups, and we were not able to evaluate the difference of compliance between the T&E and PRN groups. However, our results reflect the true conditions that physicians might encounter in their daily practice in a real-life setting; hence, it will serve as an important reference for the real-world treatment outcomes of patients with DME.

In conclusion, this study demonstrated the superior treatment response of the T&E regimen of ranibizumab in treating DME with better visual and anatomical outcomes compared to the PRN regimen in a real-world setting. The 2-year results of our study confirmed the usefulness of the T&E regimen and decreased the need for both injections and clinic visits in the second year.

## Data Availability Statement

The raw data supporting the conclusions of this article will be made available by the authors, without undue reservation.

## Ethics Statement

The studies involving human participants were reviewed and approved by the National Taiwan University Hospital Research Ethics Committee (No.: 201811023RIFD). Written informed consent for participation was not required for this study in accordance with the national legislation and the institutional requirements.

## Author Contributions

Y-TH contributed to conception and design of the study. T-TL and Y-TH organized the database. T-TL performed the statistical analysis and wrote the first draft of the manuscript. T-CC, C-HY, C-MY, T-CH, and Y-TH wrote sections of the manuscript. All authors contributed to manuscript revision, read, and approved the submitted version.

## Funding

Novartis Taiwan sponsored this study. The funding agreement ensured the authors' independence in designing the study, interpreting the data, writing, and publishing the report.

## Conflict of Interest

T-TL, T-CC, C-HY, C-MY, T-CH, and Y-TH received honoraria for speeches from Novartis Taiwan.

## Publisher's Note

All claims expressed in this article are solely those of the authors and do not necessarily represent those of their affiliated organizations, or those of the publisher, the editors and the reviewers. Any product that may be evaluated in this article, or claim that may be made by its manufacturer, is not guaranteed or endorsed by the publisher.
