# Supplementary material for: Treat-and-Extend vs. Pro Re Nata Regimen of Ranibizumab for Diabetic Macular Edema—A Two-Year Matched Comparative Study
Source: Front Med (Lausanne). 2022 Jan 25;8:781421. doi: 10.3389/fmed.2021.781421 (PMC8821911; doi:10.3389/fmed.2021.781421)
Supplement: Supplementary file 1 [file Table_1.pdf]

Supplementary Table 1. Two different treatment regimens for anti-vascular endothelial growth factor therapy in patients with diabetic macular edema.

**PRN regimen** (BCVA and OCT examined at every clinic visit)	
**Injection**	**Follow-up only**
● BCVA loss ≥ 5 ETDRS letters<br>OR<br>● New or increased SRF and/or IRC<br>OR<br>● CFT increase ≥ 100 μm	● BCVA loss < 5 ETDRS letters<br>AND<br>● No SRF and IRC, or stable IRC over 2 consecutive treatment visits after the loading phase<br>AND<br>● CFT increase < 100 μm
**T&E regimen** (BCVA and OCT examined at every clinic visit)	
**Shorten** (- 4 weeks)<br>Minimum interval: 4 weeks	**Extend** (+ 4 weeks)<br>Maximum interval: 24 weeks
● BCVA loss ≥ 5 ETDRS letters<br>OR<br>● New or increased SRF and/or IRC<br>OR<br>● CFT increase ≥ 100 μm	● BCVA loss < 5 ETDRS letters<br>AND<br>● No SRF and IRC, or stable IRC over 2 consecutive treatment visits after the loading phase<br>AND<br>● CFT increase < 100 μm

BCVA, best-corrected visual acuity; CFT, central foveal thickness; ETDRS, Early Treatment Diabetic Retinopathy Study; IRC, intraretinal cyst; OCT, optical coherence tomography; PRN, pro re nata; SRF, subretinal fluid; T&E, treat-and-extend.
